# Association between preoperative sarcopenia and postoperative delirium in older patients undergoing gastrointestinal cancer surgery

**DOI:** 10.3389/fnagi.2024.1416569

**Published:** 2024-07-31

**Authors:** Bo Dong, Dongdong Yu, Huanhuan Zhang, Pan Li, Yi Li, Chong Li, Jianli Li

**Affiliations:** ^1^Department of Anesthesiology, Hebei General Hospital, Shijiazhuang, Hebei, China; ^2^Graduate Faculty, Hebei North University, Zhangjiakou, China

**Keywords:** older patients, risk factors, sarcopenia, postoperative delirium, gastrointestinal cancer surgery

## Abstract

**Background:**

Postoperative delirium (POD) is a usual neurological complication, often leading to poor prognoses. Early identification of high-risk patients is crucial for preventing POD. Sarcopenia is an age-related geriatric syndrome characterized by the loss of skeletal muscle mass and function, and previous studies indicated that preoperative low muscle mass might be a predictor for POD. However, the association between preoperative sarcopenia and POD remains to be fully elucidated. This study was to explore the correlation between preoperative sarcopenia and POD following gastrointestinal cancer surgery in older patients.

**Methods:**

Older patients (≥60 years) undergoing gastrointestinal cancer surgery were enrolled. Sarcopenia was defined based on the Special Interest Group on sarcopenia of the International Society of Physical and Rehabilitation Medicine (ISarcoPRM), which combined the loss of muscle mass (evaluated by ultrasound) and function (assessed by chair stand test and handgrip strength) before surgery. POD assessment was performed using the Confusion Assessment Method (CAM) or CAM for the intensive care unit (CAM-ICU) during the first 7 days after surgery or before discharge. Multivariate logistic regression analysis examined the correlation between preoperative sarcopenia and POD. Moreover, the receiver operator characteristic (ROC) curve was applied to analyze the predictive effect of the preoperative sarcopenia in POD.

**Results:**

One hundred and thirty patients were finally included, of which 43 patients presented with sarcopenia before surgery. Twenty-four patients ultimately developed POD, and the incidence was 18.5%. The results of the multivariate analyses demonstrated that preoperative sarcopenia was still independently associated with POD after adjusting for age ≥70 years, preoperative Mini-Mental State Examination score, and intraoperative blood transfusion. The area under the ROC curve of preoperative sarcopenia in predicting POD was 0.680 (95% confidence interval 0.557–0.804).

**Conclusion:**

Preoperative sarcopenia defined by ISarcoPRM criteria was independently associated with POD in geriatric patients after gastrointestinal cancer surgery.

## 1 Introduction

With the rapidly increasing prevalence of cancer, the global cancer cases requiring surgery are expected to be 13.8 million in 2040, a 52% rise from 2018 (Perera et al., 2021). It is estimated that gastrointestinal cancer surgery will account for 18% of all cancer surgery in 2040 (Perera et al., 2021). Although enhanced recovery after surgery (ERSA) was widely implemented in gastrointestinal cancer surgery, postoperative complications remained common (Feng et al., 2023). Postoperative delirium (POD) is a usual neurocogenitive complication characterized by acute onset of deficits in attention and other aspects of cognition (Wilson et al., 2020; Bellelli et al., 2021). It is reported that approximately 11.1% to 45.6% of patients experience POD, depending on the different methods of assessment, surgery types, and sample size (Ho et al., 2021). In addition, POD could lead to various unfavorable events, including prolonged hospital stay, higher rate of unplanned non-home discharge, as well as increased risk of surgical complications and mortality (Bellelli et al., 2018; Yan et al., 2023). Therefore, exploring effective strategies to prevent and treat POD is clinically imperative. POD could often be triggered when predisposing risk factors interact with precipitating risk factors (Oh and Park, 2019). Fortunately, it is estimated that up to 40% of POD cases could be prevented by early identifying its modifiable predisposing risk factors, albeit the exact pathophysiologic mechanisms of POD remain obscure (Schenning and Deiner, 2015). Several modifiable predisposing risk factors were identified, such as frailty, malnutrition, low albumin concentration, anticholinergic drug burden related to polypharmacy, and preoperative anemia (Pasina et al., 2019; Hughes et al., 2020).

Sarcopenia, characterized by the loss of skeletal muscle mass and function, is an age-related geriatric syndrome (Cruz-Jentoft and Sayer, 2019), which is deemed as the biologic marker of frailty (Cruz-Jentoft et al., 2019). The global prevalence of sarcopenia is more frequent with increasing age, ranging from 10 to 27% in people over 60 years (Petermann-Rocha et al., 2022). Although sarcopenia has attracted increasing attention from clinical physicians and researchers and is regarded as a muscle disease by the International Classification of Disease 10th edition, it still lacks a generally accepted clinical definition or diagnostic criteria (Falcon and Harris-Love, 2017). Several international consensus guidelines proposed diagnostic algorithms for sarcopenia, most of which used combinations of loss of muscle mass and function (Sayer and Cruz-Jentoft, 2022). Skeletal muscle mass, as one of the primary parameters of sarcopenia, could be measured by computed tomography (CT), ultrasound, magnetic resonance imaging (MRI), and dual-energy X-ray absorptiometry (DXA) (Chianca et al., 2022). In particular, CT is widely used in routine clinical examinations and thus increasing retrospective studies of sarcopenia based on previous CT findings were conducted (Vogele et al., 2023). Additionally, the value of CT was also reported in sarcopenia patients with cancer and hip fractures (Li et al., 2024). A multitude of studies reported that low skeletal muscle mass determined by CT was significantly associated with increased hospital stay, surgical complications, and lower survival rate (Xiao et al., 2022; Knoedler et al., 2023). In addition, several studies indicated that CT-determined low skeletal muscle mass was related to increased risk of POD (Mosk et al., 2018; Hirase et al., 2021; van der Zanden et al., 2021). However, it is important to point out that CT is radioactive, expensive, and not routinely performed in clinical practice. Recently, muscle ultrasound is deemed as a promising tool for evaluating muscle mass in diagnosing sarcopenia due to its radiation-free, inexpensiveness, and ease of accessibility (Fu et al., 2023). Although a recent study regarded the preoperative low muscle mass measured by ultrasound as a predictor for the development of POD (Canales et al., 2022), it was noteworthy that sarcopenia diagnosis required low muscle mass plus low muscle function according to the international consensus guidelines (Sayer and Cruz-Jentoft, 2022). In 2021, the Special Interest Group on sarcopenia of the International Society of Physical and Rehabilitation Medicine (ISarcoPRM) developed novel diagnostic criteria based on combinations of loss of muscle mass (evaluated by ultrasound) and function (assessed by handgrip strength or chair stand test) (Kara et al., 2021). However, to our knowledge, the association between sarcopenia defined by ISarcoPRM and POD has not been investigated so far.

We therefore aimed to explore the correlation between sarcopenia defined by ISarcoPRM and POD in geriatric patients undergoing gastrointestinal cancer surgery.

## 2 Materials and methods

### 2.1 Study design and population

This prospective cohort study was approved by the Medical Ethics Committee of Hebei General Hospital (approval No.2023-85) and registered at clinicaltrials.gov (No. ChiCTR2300072629). The study adhered to the Declaration of Helsinki and all patients signed informed consent.

From June 2023 to November 2023, patients who were scheduled for gastrointestinal cancer surgery in Hebei General Hospital were eligible for inclusion. The inclusion criteria were : (1) age ≥ 60 years; (2) undergoing laparoscopic gastric or colorectal cancer surgery; and (3) American Society of Anesthesiologists (ASA) physical status class II-III. The exclusion criteria were: (1) preexisting delirium, dementia, or severe mental disorders; (2) communication or cooperation difficulty; (3) intraoperative conversion to open surgery or not accepting surgery; (4) not completing the sarcopenia or POD assessment.

### 2.2 Data collection

We used a structured questionnaire to collect preoperative, intraoperative, and postoperative variables. The preoperative data included age, sex, ASA physical status, educational level, Body Mass Index (BMI), preoperative Mini-Mental State Examination (MMSE) score, types of cancer, preoperative comorbidities, smoking and drinking history, and Charlson Comorbidity Index (CCI), etc. Preoperative anxiety or depression was assessed using the Hospital Anxiety and Depression Scale (HADS) (Segernäs et al., 2022). Preoperative nutrition status was determined by mini nutritional assessment short form (MNA-SF; score range: 0–14: scores < 12 indicate risk of malnutrition) (Kaiser et al., 2009). In addition, we also collected some preoperative laboratory data such as hemoglobin, hematocrit, blood type, white blood cell count, platelet count, creatinine, blood glucose, serum albumin, sodium, potassium, as well as total cholesterol, etc. The intraoperative data contained duration of operation and anesthesia, intraoperative blood loss and transfusion, patient-controlled intravenous analgesia (PCIA), etc. The postoperative data were collected as follows: length of stay in hospital after surgery, Intensive care unit (ICU) admission, and all-cause 30-day mortality.

### 2.3 Sarcopenia assessment

All patients completed the assessment of sarcopenia based on the ISarcoPRM algorithm 1 day before surgery, including loss of muscle mass and function (Kara et al., 2021). Muscle mass was measured by the Philips CX50 Color Doppler Ultrasound System with a probe frequency of 3 to 12 MHz (Kara et al., 2020). The ultrasound images of the anterior thigh muscle were obtained at the midpoint between the anterior superior iliac spine and the superior border of the patella when patients lay in the supine position. During the measurements, we used plenty of gel to avoid any compression. The anterior thigh muscle thickness was determined as the distance between the cortex of the femur and the most superficial muscular fascia. Three consecutive images of the anterior thigh muscle were acquired, and the measurements were averaged. To eliminate the potential effects of body mass and height, the Sonographic Thigh Adjustment Ratio (STAR) was calculated as the anterior thigh muscle thickness divided by BMI. The same anesthesiologist, an expert in musculoskeletal ultrasound, acquired all images and performed all measurements. Besides, handgrip strength and chair stand test (CST) were applied to assess muscle function (Pinheiro et al., 2016). Handgrip strength was assessed by a Camry digital hand dynamometer with the dominant hand, and measurements were repeated 3 times and then averaged. For the CST, patients were required to cross their arms over their chest and rise from a chair 5 times as fast as possible, and the time was recorded. According to ISarcoPRM definitions, the presence of sarcopenia was determined if low muscle mass (according to STAR values < 1.0 for females and <1.4 for males) combined with low handgrip strength (<19 kg for females and <32 kg for males), and/or CST time ( ≥ 12 s).

### 2.4 Postoperative delirium assessment

A trained anesthesiologist evaluated POD two times per day (before 10 am and after 5 pm) within 7 postoperative days or before hospital discharge using the Confusion Assessment Method (CAM) (Inouye et al., 1990) or the CAM for the intensive care unit (CAM-ICU) (Ely et al., 2001). For non-intubated patients, CAM was used to diagnose POD. For intubated patients admitted to the ICU postoperatively, the level of sedation was measured first via the Richmond Agitation and Sedation Scale, and then CAM-ICU was used to diagnose POD. Both CAM and CAM-ICU were based on four features : (1) an acute fluctuation in mental status, (2) inattention, (3) an altered level of consciousness, and (4) disordered thinking. POD was diagnosed when (1) and (2) were present together with either (3) or (4). Besides, the subtypes and overall duration of POD were also measured. The subtypes of POD were determined using the RASS score: hypoactive type, RASS score < 0; hyperactive type, RASS score > 0; and mixed type, the hypoactive and hyperactive types occurred alternately (Peterson et al., 2006).

### 2.5 Sample size calculation

The minimum sample size of this study was calculated using the logistic regression models events per variable sample size calculation method, and at least 6–10 events were needed per variable (Peduzzi et al., 1996). According to the reported prevalence of POD after gastrointestinal cancer surgery of 25.8% (Liu et al., 2023) and a 20% dropout rate, at least 116 patients were needed to allow 4 variables in the final multivariable model.

### 2.6 Statistical analysis

Quantitative data were expressed as mean and standard deviation or as median with interquartile ranges, and compared with independent sample t-test for normal distribution or Mann–Whitney U test for non-normal distribution. Qualitative data were reported as numbers and percentages, and compared with chi-square test or Fisher test. The multicollinearity among covariates was examined using the tolerance (Tol) and variance inflation factor (VIF). Initially, all variables were assessed for association with POD by univariate regression analysis. Furthermore, variables with a *P* < 0.05 in univariable analysis were put into multivariate analysis to identify the independent risk factors for POD. The multivariate analysis results were presented as odds ratio (OR) and 95% confidence interval (CI). In addition, the receiver operator characteristic (ROC) curve was applied to analyze the predictive effect of preoperative sarcopenia in POD. *P* < 0.05 was deemed as statistical significance. All data were analyzed by SPSS version 26.0 (SPSS Inc., Chicago, IL, USA).

## 3 Results

**FIGURE 1:**
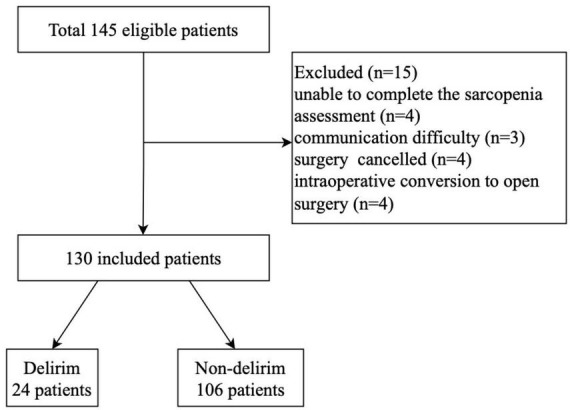
Flow chart of study population.

From June 2023 to November 2023, there were 145 patients (≥60 years) who underwent laparoscopic gastric or colorectal cancer surgery eligible for inclusion. Fifteen patients were excluded for several reasons as follows: unable to complete the sarcopenia assessment (*n* = 4), had communication difficulty (*n* = 3), surgery canceled (*n* = 4), and intraoperative conversion to open surgery (*n* = 4). Thus, the remaining 130 patients were finally included (Figure 1).

### 3.1 Patients’ characteristics

Table 1 showed characteristics of patients stratified by sarcopenia, with a mean age of 73 (66–77) years old and 61.5% male patients. The prevalence of sarcopenia was approximately 33.1% (43 patients). Sarcopenia patients were generally older, had a higher prevalence of hypertension, diabetes mellitus, and malnutrition, higher baseline CCI, and lower preoperative MMSE scores compared with non-sarcopenia patients. In addition, sarcopenia patients received more preoperative chemotherapy treatment than non-sarcopenia patients. To compare the prevalence of POD in sarcopenia patients with preoperative chemotherapy and without preoperative chemotherapy, we carried out an in-depth study in 43 sarcopenia patients and the results showed that the incidence of POD was 41.7% (5/12) in sarcopenia patients with preoperative chemotherapy and 32.2% (10/31) in sarcopenia patients without preoperative chemotherapy, but the difference was not statistically significant (*P* > 0.05), which might be related to the insufficient sample size in this study. In contrast, there were no differences in sex, educational level, coronary heart disease, smoking history, drinking history, chronic obstructive pulmonary disease, anxiety, depression, cerebrovascular disease, and preoperative radiotherapy treatment.

**TABLE 1:** Demographic characteristics of the patients with and without preoperative sarcopenia.

Variable	All patients (*n* = 130)	Sarcopenia (*n* = 43)	Non-sarcopenia (*n* = 87)	*P-*value
Age (years)	73 (66–77)	74 (70–80)	69 (62–75)	0.001
Male gender, *n* (%)	80 (61.5)	31 (72.1)	49 (56.3)	0.082
Body mass index (kg/m^2^)	24.18 ± 3.20	23.74 ± 3.65	24.39 ± 2.96	0.005
Educational level, *n* (%)				0.728
Low degree (illiteracy and primary school)	70 (53.8)	25 (58.1)	45 (51.7)	
Medium degree (middle and senior school)	43 (33.1)	11 (25.6)	32 (36.8)	
High degree (college and above)	17 (13.1)	7 (16.3)	10 (11.5)	
Smoking history, *n* (%)	42 (32.3)	13 (30.2)	29 (33.3)	0.722
Drinking history, *n* (%)	15 (11.5)	3 (7.0)	12 (13.8)	0.252
Comorbidities, *n* (%)				
Hypertension	70 (53.8)	29 (67.4)	41 (47.1)	0.029
Diabetes mellitus	35 (26.9)	17 (39.5)	18 (20.7)	0.023
Coronary heart disease	15 (11.5)	6 (14.0)	9 (10.3)	0.545
Chronic obstructive pulmonary disease	15 (11.5)	4 (9.3)	11 (12.6)	0.575
Cerebrovascular disease	9 (6.9)	4 (9.3)	5 (5.7)	0.452
ASA physical status, *n* (%)				0.023
Class II	58 (45.3)	13 (31.0)	45 (52.3)	
Class III	70 (54.7)	29 (69.0)	41 (47.7)	
Pre-operative MMSE scores	28 (26–28)	28 (27–29)	27 (26–28)	0.016
Anxiety, *n* (%)	15 (11.5)	4 (9.3)	11 (12.6)	0.575
Depression, *n* (%)	10 (7.7)	2 (4.7)	8 (9.2)	0.360
CCI	1.0 (0.0–2.0)	2.0 (0.0–2.0)	1.0 (0.0–1.0)	0.015
Preoperative treatment, *n* (%)				
Chemotherapy	21 (16.2)	12 (27.9)	9 (10.3)	0.010
Radiotherapy	2 (1.5)	1 (2.3)	1 (1.1)	0.608
Preoperative nutrition status (MNA-SF), *n* (%)				0.005
normal (12–14)	93 (71.5)	24 (55.8)	69 (79.3)	
risk of malnutrition (<12)	37 (28.5)	19 (44.2)	18 (20.7)	

ASA, American Society of Anesthesiologists; MMSE, Mini-Mental State Examination; CCI, Charlson Comorbidity Index; MNA-SF, Mini Nutritional Assessment-Short Form.

### 3.2 Clinical outcomes of patients

The incidence of POD in the elderly gastrointestinal cancer surgery was 18.5% (24/130) and the mean duration of POD was 2 (1–2) days. There were 7 cases (29.2%) of hypoactive delirium, 14 cases (58.3%) of hyperactive delirium, and 3 cases (12.5%) of mixed delirium. There was a statistically higher prevalence in sarcopenia patients compared to non-sarcopenia patients (32.6% vs. 11.5%, *P* = 0.001). In addition, the in-hospital length of stay after surgery was longer in the sarcopenia group compared to the non-sarcopenia group (*P* = 0.015). No differences were observed in the ICU admission and all-cause 30-day mortality between the sarcopenia and non-sarcopenia group, as presented in Table 2.

**TABLE 2:** Postoperative outcomes in sarcopenia and non-sarcopenia patients.

Variable	All patients (*n* = 130)	Sarcopenia (*n* = 43)	Non-sarcopenia (*n* = 87)	*P-*value
Delirium within 7 days	24 (18.5)	15 (34.9)	9 (10.3)	0.001
ICU admission	11 (8.5)	6 (14.0)	5 (5.7)	0.114
Length of stay in hospital after surgery (day)	10 (8–12)	11 (9–14)	10 (8–11)	0.015
All-cause 30-day mortality	2 (1.5)	1 (2.3)	1 (1.1)	0.608

ICU, Intensive Care Unit.

### 3.3 Multicollinearity among all independent variables

Our results, as shown in Supplementary Table 1, indicated that there was no severe collinearity among all independent variables (all Tol > 0.1, VIF < 10).

### 3.4 The association between preoperative sarcopenia and POD

**FIGURE 2:**
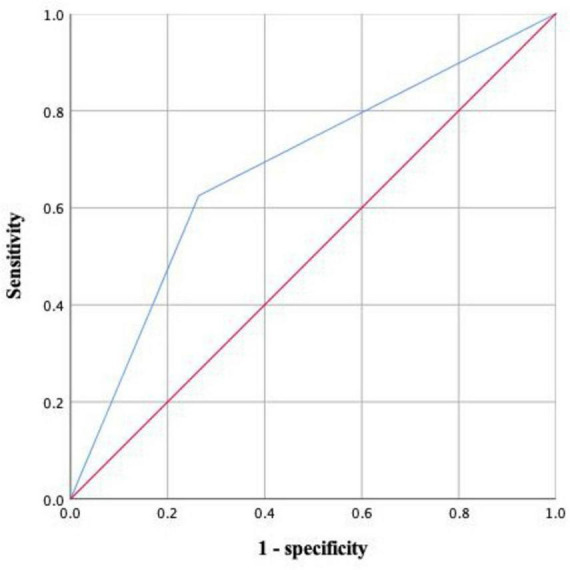
The predictive value of preoperative sarcopenia for POD by ROC analysis.

Initially, the results of the univariate logistic regression analysis showed that age ≥ 70 years, preoperative MMSE score (continuous), serum albumin (continuous), malnutrition (categorical), intraoperative blood transfusion (categorical), as well as sarcopenia were deemed as the potential risk factors for POD. Furthermore, the multivariate logistic regression analysis results showed that preoperative sarcopenia (OR: 4.620; 95% CI:1.523–14.018; *P* = 0.007) was still an independent risk factor for POD after adjusting for age ≥ 70 years (OR: 5.647; 95% CI: 1.276–24.981; *P* = 0.023), preoperative Mini-Mental State Examination scores (OR: 0.509; 95% CI: 0.303–0.855; *P* = 0.011), and intraoperative blood transfusion (OR: 4.806; 95% CI: 1.037–22.280; *P* = 0.045), as demonstrated in Table 3. As presented in Figure 2, the area under the ROC curve of preoperative sarcopenia in predicting POD was 0.680 (95% CI 0.557–0.804).

**TABLE 3:** Risk factors for postoperative delirium by univariate and multivariate logistic regression analyses.

Variable	Univariate analyses		Multivariate analysis	
	**OR (95% CI)**	***P*-value**	**OR (95% CI)**	***P-*value**
Age ≥ 70 years	6.491 (1.826–23.073)	0.004	5.647 (1.276–24.981)	0.023
Sarcopenia	4.643 (1.827–11.796)	0.001	4.620 (1.523–14.018)	0.007
Cerebrovascular disease	4.040 (0.997–16.376)	0.051		
Pre-operative MMSE scores	0.517 (0.332–0.803)	0.003	0.509 (0.303–0.855)	0.011
CCI	1.528 (0.975–2.394)	0.064		
eGFR (ml/min/1.73 m^2^)	0.971 (0.934–1.010)	0.148		
Serum albumin (g/L)	0.877 (0.784–0.981)	0.021		
**Preoperative nutrition status (MNA-SF)**				
Normal (12–14)				
Risk of malnutrition (<12)	2.604 (1.041–6.512)	0.041		
Intraoperative blood transfusion	5.044 (1.617–15.737)	0.005	4.806 (1.037–22.280)	0.045

ASA, American Society of Anesthesiologists; MMSE, Mini-Mental State Examination; CCI, Charlson Comorbidity Index; MNA-SF, Mini Nutritional Assessment-Short Form; CI, confidence interval; OR, Odds Ratio; eGFR, estimated glomerular filtration rate.

## 4 Discussion

POD is a frequent neurological complication after gastrointestinal cancer surgery and often results in high morbidity and mortality (Wang et al., 2023). Given these negative outcomes, it is crucial to identify modifiable risk factors for POD and implement appropriate interventions to avoid POD. As far as we know, this was the first prospective cohort study to explore the relationship between sarcopenia defined by ISarcoPRM and POD after gastrointestinal cancer surgery. Our results showed strong evidence of a connection between sarcopenia and POD in older patients following gastrointestinal cancer surgery.

The incidence of POD varies widely across surgery types, being 14% for colorectal surgery, 20% for orthopedic procedures, and 30% for cardiac surgery according to a recent study (Igwe et al., 2023). In our study, the POD incidence was 18.5% after gastrointestinal cancer surgery, consistent with a previous study (Lin et al., 2021). However, the POD rate was lower than in a previous study with an incidence of 33.5% (Sugi et al., 2023), which might be associated with the lower mean age of patients in our study. Additionally, it was reported that using a laparoscopic approach could reduce the occurrence of POD after major abdominal surgery (Ito et al., 2019). In our study, we only enrolled patients undergoing laparoscopic surgery for gastric or colorectal cancer, which might also contribute to a relatively lower incidence of POD.

Although the specific pathophysiologic mechanism for POD remains obscure and the most efficient prevention strategies have yet to be established, identifying its associated risk factors might be the first step to preventing POD. As previous studies reported, several well-recognized risk factors could induce POD after gastrointestinal surgery, such as advanced age, lower preoperative MMSE scores, preoperative C-reactive protein levels, perioperative blood transfusion, and longer operation time (Sugi et al., 2023; Wang et al., 2023). Likewise, our results showed that the independent risk factors for POD after gastrointestinal cancer surgery included age ≥ 70 years, lower preoperative MMSE score, and intraoperative blood transfusion. The older patients were more likely to experience POD, which might be related to the simultaneous presence of numerous risk factors for POD, including more comorbidities, polypharmacy, and cognitive impairment in older patients (Poeran et al., 2020). The MMSE score is regarded as a validated screening test to assess cognitive status, and lower scores indicate worse cognitive function (Mowla and Zandi, 2006). So, patients with lower preoperative MMSE scores should be of great concern to clinicians. Not surprisingly, intraoperative blood transfusion is relevant to the development of POD due to the increased inflammatory response and impaired innate immunity (Aldecoa et al., 2023). In addition, a previous study suggested that serum album was independently associated with POD in colorectal surgery (Lee and Lim, 2020). However, our results showed that serum albumin was not an independent risk factor for POD after gastrointestinal cancer surgery, which might be related to the effect of sarcopenia on modifying the serum albumin/POD association (Brown et al., 2016).

Noticeably, the association between preoperative sarcopenia and POD, independent of age ≥ 70 years, preoperative MMSE score, and intraoperative blood transfusion, was an important finding in the current study. Sarcopenia is known to be more prevalent in older patients with gastrointestinal cancer due to inflammation, physical inactivity, and malnutrition (Williams et al., 2019). A previous meta-analysis reported that the prevalence of sarcopenia ranged from 6.8 to 35.9% after surgery for gastrointestinal cancer (Pipek et al., 2020). In our study, the prevalence of sarcopenia was approximately 33.1%, which was similar to a previous study with a prevalence of 38.57% after gastrointestinal or hepato-pancreatico-biliary cancer surgery (Nagarajan et al., 2023). However, some studies reported a lower prevalence of sarcopenia compared to our study, such as 21.07% by Ding et al. (2022) and 21.2% by Erkul et al. (2022), which might be attributed to differences in diagnostic criteria, study populations, and sample size.

Several studies indicated that preoperative sarcopenia was correlated with poor overall survival and disease-free survival after cancer surgery (He et al., 2023; Park et al., 2023). However, only a few studies investigated the potential correlation between low muscle mass and POD. A previous study by Makiguchi et al. (2020) indicated that low muscle mass significantly increased the risk of hypoactive and mixed-type POD in patients undergoing oral cancer surgery. In addition, a recent meta-analysis suggested that hospitalized older patients with low skeletal muscle mass were at increased risk of delirium (Shen et al., 2023). However, these studies only explored the effect of preoperative low muscle mass rather than sarcopenia on POD. The revised European Working Group on Sarcopenia in Older People emphasized that the loss of muscle function was a crucial characteristic of sarcopenia besides muscle mass (Cruz-Jentoft et al., 2019), and a study demonstrated a significant association between low preoperative hand grip strength and POD after cardiovascular surgery (Kotani et al., 2023). Our results showed that preoperative sarcopenia was independent risk factor for POD, which confirmed and extended prior studies examining the correlation between preoperative sarcopenia and POD. While ROC curve analysis showed that preoperative sarcopenia had a modest predictive ability for POD with an area under the curve of 0.680, this study still indicated that routine preoperative screening for sarcopenia was beneficial in identifying the occurrence of POD. Importantly, sarcopenia was assessed using the ISarcoPRM algorithm based on combinations of loss of muscle mass and muscle function in the current study. Moreover, it should be noted that we used ultrasound to assess the muscle mass, which was strongly correlated with MRI-, DXA-, and CT-based muscle measurements and had the advantage of simplicity and reproducibility in clinical practice (Perkisas et al., 2021). However, small variations always occurred during muscle measurement due to various reasons, thus we used the mean value of three measurements for thigh muscle to reduce errors.

Currently, despite the discovery of new targets and the development of new drugs, nonpharmacological therapies are considered the basis for the prevention and treatment of sarcopenia, including dietary changes, nutritional supplements, or treating underlying inflammatory bowel disease were used to counteract sarcopenia (Liu et al., 2024). Our results showed that preoperative sarcopenia was independently associated with POD in older patients after gastrointestinal cancer surgery. Given the adverse prognosis of POD, further research is needed to explore whether some nonpharmacological interventions for sarcopenia could reduce the risk of POD.

Although the underlying mechanism for the correction between preoperative sarcopenia and POD is unclear, there are several reasons why POD is likely to develop in patients with sarcopenia. First, predisposing factors underlying sarcopenia are deemed as common risk factors for POD, such as malnutrition, physical inactivity, and cognitive impairment (Hughes et al., 2020). Second, a recent animal study found that preoperative low skeletal muscle mass was related to an increased risk of perioperative neurocognitive disorder through the reduction of brain-derived neurotrophic factor (Nemoto et al., 2022). Third, sarcopenia and POD might share common pathophysiological mechanisms, including inflammation, oxidative stress, and impairment of neuroendocrine regulation (Cruz-Jentoft et al., 2019).

Several limitations of our study should be addressed. Firstly, we conducted this prospective study with a relatively small sample size in a single-center institution. Future large-sample, multi-center prospective cohort studies should be performed to validate our findings. Secondly, due to the fact that our study population was restricted to gastrointestinal cancer surgery patients, our findings might not be generalized to other types of surgery patients. Lastly, although we attempted to adjust for some potential confounders in binary logistic regression analysis, some other risk factors for POD, such as frailty, perioperative sleep disturbance, intraoperative hypothermia, preoperative multiple medications use, etc, were not included in our analyses.

## 5 Conclusion

In the current study, our results suggested that preoperative sarcopenia might be a modifiable risk factor for POD following gastrointestinal cancer surgery in geriatric patients. Further research is needed to elucidate the underlying mechanisms of this association and explore whether targeted nutrition and exercise-based interventions for sarcopenia could reduce the risk of POD.

## Data availability statement

The raw data supporting the conclusions of this article will be made available by the authors, without undue reservation.

## Ethics statement

The studies involving humans were approved by the Medical Ethics Committee of Hebei General Hospital (approval No.2023-85). The studies were conducted in accordance with the local legislation and institutional requirements. The participants provided their written informed consent to participate in this study.

## Author contributions

BD: Writing – original draft. DY: Data curation, Writing – review and editing. HZ: Formal analysis, Writing – review and editing. PL: Validation, Writing – review and editing. YL: Methodology, Writing – review and editing. CL: Formal analysis, Writing – review and editing. JL: Writing – original draft.

## References

[B1] Aldecoa C. Bettelli G. Bilotta F. Sanders R. D. Aceto P. Audisio R. (2023). Update of the European society of anaesthesiology and intensive care medicine evidence-based and consensus-based guideline on postoperative delirium in adult patients. *Eur. J. Anaesthesiol.* 41 81–108. 10.1097/EJA.0000000000001876 37599617 PMC10763721

[B2] Bellelli G. Brathwaite J. S. Mazzola P. (2021). Delirium: A marker of vulnerability in older people. *Front. Aging Neurosci.* 13:626127. 10.3389/fnagi.2021.626127 33994990 PMC8119654

[B3] Bellelli G. Carnevali L. Corsi M. Morandi A. Zambon A. Mazzola P. (2018). The impact of psychomotor subtypes and duration of delirium on 6-month mortality in hip-fractured elderly patients. *Int. J. Geriatr. Psychiatry.* 10.1002/gps.4914 [Epub ahead of print]. 29851194

[B4] Brown C. H. T. Max L. Laflam A. Kirk L. Gross A. Arora R. (2016). The association between preoperative frailty and postoperative delirium after cardiac surgery. *Anesth. Analg.* 123 430–435. 10.1213/ANE.0000000000001271 27096563 PMC5406128

[B5] Canales C. Mazor E. Coy H. Grogan T. R. Duval V. Raman S. (2022). Preoperative point-of-care ultrasound to identify frailty and predict postoperative outcomes: A diagnostic accuracy study. *Anesthesiology* 136 268–278. 10.1097/ALN.0000000000004064 34851395 PMC9843825

[B6] Chianca V. Albano D. Messina C. Gitto S. Ruffo G. Guarino S. (2022). Sarcopenia: Imaging assessment and clinical application. *Abdom. Radiol.* 47 3205–3216. 10.1007/s00261-021-03294-3 34687326 PMC8536908

[B7] Cruz-Jentoft A. J. Sayer A. A. (2019). Sarcopenia. *Lancet* 393 2636–2646. 10.1016/S0140-6736(19)31138-9 31171417

[B8] Cruz-Jentoft A. J. Bahat G. Bauer J. Boirie Y. Bruyère O. Cederholm T. (2019). Sarcopenia: Revised European consensus on definition and diagnosis. *Age Ageing* 48:601. 10.1093/ageing/afz046 31081853 PMC6593317

[B9] Ding P. Guo H. Sun C. Chen S. Yang P. Tian Y. (2022). Serum creatinine/cystatin C ratio is a systemic marker of sarcopenia in patients with gastrointestinal stromal tumours. *Front. Nutr.* 9:963265. 10.3389/fnut.2022.963265 36118766 PMC9478187

[B10] Ely E. W. Margolin R. Francis J. May L. Truman B. Dittus R. (2001). Evaluation of delirium in critically ill patients: Validation of the confusion assessment method for the intensive care unit (Cam-Icu). *Crit. Care Med.* 29 1370–1379. 10.1097/00003246-200107000-00012 11445689

[B11] Erkul O. Cekic A. B. Cansu A. Yildirim R. Guner A. (2022). Effects of sarcopenia on postoperative outcomes in patients who underwent gastrectomy for gastric cancer. *J. Surg. Res.* 274 196–206. 10.1016/j.jss.2021.12.051 35183030

[B12] Falcon L. J. Harris-Love M. O. (2017). Sarcopenia and the New ICD-10-CM Code: Screening, staging, and diagnosis considerations. *Fed. Pract.* 34 24–32. 28867927 PMC5576154

[B13] Feng J. Y. Wang S. F. Yan J. (2023). The application of enhanced recovery after surgery for gastrectomy and colorectal resection: A systematic review and meta-analysis. *J. Laparoendosc. Adv. Surg. Tech. A* 33 586–595. 10.1089/lap.2023.0036 37130316

[B14] Fu H. Wang L. Zhang W. Lu J. Yang M. (2023). Diagnostic test accuracy of ultrasound for sarcopenia diagnosis: A systematic review and meta-analysis. *J. Cachexia Sarcopenia Muscle* 14 57–70. 10.1002/jcsm.13149 36513380 PMC9891970

[B15] He J. Luo W. Huang Y. Song L. Mei Y. (2023). Sarcopenia as a prognostic indicator in colorectal cancer: An updated meta-analysis. *Front. Oncol.* 13:1247341. 10.3389/fonc.2023.1247341 37965475 PMC10642225

[B16] Hirase T. Haghshenas V. Bratescu R. Dong D. Kuo P. H. Rashid A. (2021). Sarcopenia predicts perioperative adverse events following complex revision surgery for the thoracolumbar spine. *Spine J.* 21 1001–1009. 10.1016/j.spinee.2021.02.001 33561547

[B17] Ho M. H. Nealon J. Igwe E. Traynor V. Chang H. R. Chen K. H. (2021). Postoperative delirium in older patients: A systematic review of assessment and incidence of postoperative delirium. *Worldviews Evid. Based Nurs.* 18 290–301. 10.1111/wvn.12536 34482593

[B18] Hughes C. G. Boncyk C. S. Culley D. J. Fleisher L. A. Leung J. M. Mcdonagh D. L. (2020). American society for enhanced recovery and perioperative quality initiative joint consensus statement on postoperative delirium prevention. *Anesth. Analg.* 130 1572–1590. 10.1213/ANE.0000000000004641 32022748 PMC7379173

[B19] Igwe E. O. Nealon J. O’shaughnessy P. Bowden A. Chang H. R. Ho M. H. (2023). Incidence of postoperative delirium in older adults undergoing surgical procedures: A systematic literature review and meta-analysis. *Worldviews Evid. Based Nurs.* 20 220–237. 10.1111/wvn.12649 37128953

[B20] Inouye S. K. Van Dyck C. H. Alessi C. A. Balkin S. Siegal A. P. Horwitz R. I. (1990). Clarifying confusion: The confusion assessment method. A new method for detection of delirium. *Ann. Intern. Med.* 113 941–948. 10.7326/0003-4819-113-12-941 2240918

[B21] Ito K. Suka Y. Nagai M. Kawasaki K. Yamamoto M. Koike D. (2019). Lower risk of postoperative delirium using laparoscopic approach for major abdominal surgery. *Surg. Endosc.* 33 2121–2127. 10.1007/s00464-018-6483-7 30643983

[B22] KaiserM. J.BauerJ. M.RamschC.UterW.GuigozY.CederholmT. (2009). Validation of the Mini Nutritional Assessment short-form (Mna-Sf): A practical tool for identification of nutritional status. *J. Nutr. Health Aging* 13 782–788. 10.1007/s12603-009-0214-7 19812868

[B23] Kara M. Kaymak B. Ata A. M. Özkal Ö Kara Ö Baki A. (2020). STAR-sonographic thigh adjustment ratio: A golden formula for the diagnosis of sarcopenia. *Am. J. Phys. Med. Rehabil.* 99 902–908. 10.1097/PHM.0000000000001439 32941253

[B24] Kara M. Kaymak B. Frontera W. Ata A. M. Ricci V. Ekiz T. (2021). Diagnosing sarcopenia: Functional perspectives and a new algorithm from the Isarcoprm. *J. Rehabil. Med.* 53:jrm00209. 10.2340/16501977-2851 34121127 PMC8814891

[B25] Knoedler S. Schliermann R. Knoedler L. Wu M. Hansen F. J. Matar D. Y. (2023). Impact of sarcopenia on outcomes in surgical patients: A systematic review and meta-analysis. *Int. J. Surg.* 109 4238–4262. 10.1097/JS9.0000000000000688 37696253 PMC10720826

[B26] Kotani T. Ida M. Inoue S. Naito Y. Kawaguchi M. (2023). Association between preoperative hand grip strength and postoperative delirium after cardiovascular surgery: A retrospective study. *J. Clin. Med.* 12:2705. 10.3390/jcm12072705 37048787 PMC10095472

[B27] Lee S. H. Lim S. W. (2020). Risk factors for postoperative delirium after colorectal surgery: A systematic review and meta-analysis. *Int. J. Colorectal Dis.* 35 433–444. 10.1007/s00384-019-03498-6 31897646

[B28] Li L. Xia Z. Zeng X. Tang A. Wang L. Su Y. (2024). The agreement of different techniques for muscle measurement in diagnosing sarcopenia: A systematic review and meta-analysis. *Quant. Imaging Med. Surg.* 14 2177–2192. 10.21037/qims-23-1089 38545058 PMC10963824

[B29] Lin X. Wang P. Liu D. W. Guo Y. W. Xie C. H. Wang B. (2021). Intraoperative oxygen concentration and postoperative delirium after laparoscopic gastric and colorectal malignancies surgery: A randomized, double-blind, controlled trial. *Clin. Interv. Aging* 16 1085–1093. 10.2147/CIA.S311190 34163152 PMC8214551

[B30] Liu D. Wang S. Liu S. Wang Q. Che X. Wu G. (2024). Frontiers in sarcopenia: Advancements in diagnostics, molecular mechanisms, and therapeutic strategies. *Mol. Aspects Med.* 97:101270. 10.1016/j.mam.2024.101270 38583268

[B31] Liu Q. Li L. Wei J. Xie Y. (2023). Correlation and influencing factors of preoperative anxiety, postoperative pain, and delirium in elderly patients undergoing gastrointestinal cancer surgery. *BMC Anesthesiol.* 23:78. 10.1186/s12871-023-02036-w 36915054 PMC10009960

[B32] Makiguchi T. Yamaguchi T. Nakamura H. Ogawa M. Harimoto N. Shirabe K. (2020). Impact of skeletal muscle mass on postoperative delirium in patients undergoing free flap repair after oral cancer resection. *J. Plast. Surg. Hand Surg.* 54 161–166. 10.1080/2000656X.2020.1724545 32031462

[B33] Mosk C. A. Van Vugt J. L. A. De Jonge H. Witjes C. D. Buettner S. Ijzermans J. N. (2018). Low skeletal muscle mass as a risk factor for postoperative delirium in elderly patients undergoing colorectal cancer surgery. *Clin. Interv. Aging* 13 2097–2106. 10.2147/CIA.S175945 30425464 PMC6205536

[B34] Mowla A. Zandi T. (2006). Mini-mental status examination: A screening instrument for cognitive and mood disorders of elderly. *Alzheimer Dis. Assoc. Disord.* 20:124. 10.1097/01.wad.0000213812.35424.9b 16772749

[B35] Nagarajan G. Doshi P. Bardeskar N. S. Kulkarni A. Punamiya A. Tongaonkar H. (2023). Association between sarcopenia and postoperative complications in patients undergoing surgery for gastrointestinal or hepato-pancreatico-biliary cancer. *J. Surg. Oncol.* 128 682–691. 10.1002/jso.27315 37183521

[B36] Nemoto A. Goyagi T. Nemoto W. Nakagawasai O. Tan-No K. Niiyama Y. (2022). Low skeletal muscle mass is associated with perioperative neurocognitive disorder due to decreased neurogenesis in rats. *Anesth. Analg.* 134 194–203. 10.1213/ANE.0000000000005681 34347659

[B37] Oh S. T. Park J. Y. (2019). Postoperative delirium. *Korean J. Anesthesiol.* 72 4–12. 10.4097/kja.d.18.00073.1 30139213 PMC6369344

[B38] Park B. Bhat S. Xia W. Barazanchi A. W. H. Frampton C. Hill A. G. (2023). Consensus-defined sarcopenia predicts adverse outcomes after elective abdominal surgery: Meta-analysis. *BJS Open* 7:zrad065. 10.1093/bjsopen/zrad065 37542472 PMC10404004

[B39] Pasina L. Colzani L. Cortesi L. Tettamanti M. Zambon A. Nobili A. (2019). Relation between delirium and anticholinergic drug burden in a cohort of hospitalized older patients: An observational study. *Drugs Aging* 36 85–91. 10.1007/s40266-018-0612-9 30484239

[B40] Peduzzi P. Concato J. Kemper E. Holford T. R. Feinstein A. R. (1996). A simulation study of the number of events per variable in logistic regression analysis. *J. Clin. Epidemiol.* 49 1373–1379. 10.1016/S0895-4356(96)00236-3 8970487

[B41] Perera S. K. Jacob S. Wilson B. E. Ferlay J. Bray F. Sullivan R. (2021). Global demand for cancer surgery and an estimate of the optimal surgical and anaesthesia workforce between 2018 and 2040: A population-based modelling study. *Lancet Oncol.* 22 182–189. 10.1016/S1470-2045(20)30675-6 33485458

[B42] Perkisas S. Bastijns S. Baudry S. Bauer J. Beaudart C. Beckwée D. (2021). Application of ultrasound for muscle assessment in sarcopenia: 2020 Sarcus update. *Eur. Geriatr. Med.* 12 45–59. 10.1007/s41999-020-00433-9 33387359

[B43] Petermann-Rocha F. Balntzi V. Gray S. R. Lara J. Ho F. K. Pell J. P. (2022). Global prevalence of sarcopenia and severe sarcopenia: A systematic review and meta-analysis. *J. Cachexia Sarcopenia Muscle* 13 86–99. 10.1002/jcsm.12783 34816624 PMC8818604

[B44] Peterson J. F. Pun B. T. Dittus R. S. Thomason J. W. Jackson J. C. Shintani A. K. (2006). Delirium and its motoric subtypes: A study of 614 critically ill patients. *J. Am. Geriatr. Soc.* 54 479–484. 10.1111/j.1532-5415.2005.00621.x 16551316

[B45] PinheiroP. A.CarneiroJ. A.CoqueiroR. S.PereiraR.FernandesM. H. (2016). “Chair stand test” as simple tool for sarcopenia screening in elderly women. *J. Nutr. Health Aging* 20 56–59. 10.1007/s12603-016-0676-3 26728934

[B46] Pipek L. Z. Baptista C. G. Nascimento R. F. V. Taba J. V. Suzuki M. O. Do Nascimento F. S. (2020). The impact of properly diagnosed sarcopenia on postoperative outcomes after gastrointestinal surgery: A systematic review and meta-analysis. *PLoS One* 15:e0237740. 10.1371/journal.pone.0237740 32822372 PMC7446889

[B47] Poeran J. Cozowicz C. Zubizarreta N. Weinstein S. M. Deiner S. G. Leipzig R. M. (2020). Modifiable factors associated with postoperative delirium after hip fracture repair: An age-stratified retrospective cohort study. *Eur. J. Anaesthesiol.* 37 649–658. 10.1097/EJA.0000000000001197 32251149

[B48] Sayer A. A. Cruz-Jentoft A. (2022). Sarcopenia definition, diagnosis and treatment: Consensus is growing. *Age Ageing* 51 e0237740. 10.1093/ageing/afac220 36273495 PMC9588427

[B49] Schenning K. J. Deiner S. G. (2015). Postoperative delirium in the geriatric patient. *Anesthesiol. Clin.* 33 505–516. 10.1016/j.anclin.2015.05.007 26315635 PMC4555984

[B50] Segernäs A. Skoog J. Ahlgren Andersson E. Almerud, Österberg S. Thulesius H. (2022). Prediction of postoperative delirium after cardiac surgery with a quick test of cognitive speed, mini-mental state examination and hospital anxiety and depression scale. *Clin. Interv. Aging* 17 359–368. 10.2147/CIA.S350195 35400995 PMC8985827

[B51] Shen Y. Wan Q. Zhao R. Chen Y. Xia L. Wu Y. (2023). Low skeletal muscle mass and the incidence of delirium in hospitalized older patients: A systematic review and meta-analysis of observational studies. *Int. J. Clin. Pract.* 2023:4098212. 10.1155/2023/4098212 37188154 PMC10181906

[B52] Sugi T. Enomoto T. Ohara Y. Furuya K. Kitaguchi D. Moue S. (2023). Risk factors for postoperative delirium in elderly patients undergoing gastroenterological surgery: A single-center retrospective study. *Ann. Gastroenterol. Surg.* 7 832–840. 10.1002/ags3.12676 37663963 PMC10472384

[B53] van der Zanden V. Van Soolingen N. J. Viddeleer A. R. Trum J. W. Amant F. Mourits M. J. E. (2021). Low preoperative skeletal muscle density is predictive for negative postoperative outcomes in older women with ovarian cancer. *Gynecol. Oncol.* 162 360–367. 10.1016/j.ygyno.2021.05.039 34112514

[B54] Vogele D. Otto S. Sollmann N. Haggenmüller B. Wolf D. Beer M. (2023). Sarcopenia – definition, radiological diagnosis, clinical significance. *Rofo* 195 393–405. 10.1055/a-1990-0201 36630983

[B55] Wang X. Yu D. Du Y. Geng J. (2023). Risk factors of delirium after gastrointestinal surgery: A meta-analysis. *J. Clin. Nurs.* 32 3266–3276. 10.1111/jocn.16439 35791265

[B56] Williams G. R. Rier H. N. Mcdonald A. Shachar S. S. (2019). Sarcopenia & aging in cancer. *J. Geriatr. Oncol.* 10 374–377. 10.1016/j.jgo.2018.10.009 30343999 PMC6472991

[B57] Wilson J. E. Mart M. F. Cunningham C. Shehabi Y. Girard T. D. Maclullich A. M. J. (2020). Delirium. *Nat Rev. Dis. Prim.* 6:90. 10.1038/s41572-020-00223-4 33184265 PMC9012267

[B58] Xiao Y. Xiao-Yue Z. Yue W. Ruo-Tao L. Xiang-Jie L. Xing-Yuan W. (2022). Use of computed tomography for the diagnosis of surgical sarcopenia: Review of recent research advances. *Nutr. Clin. Pract.* 37 583–593. 10.1002/ncp.10847 35191086

[B59] Yan E. Veitch M. Saripella A. Alhamdah Y. Butris N. Tang-Wai D. F. (2023). Association between postoperative delirium and adverse outcomes in older surgical patients: A systematic review and meta-analysis. *J. Clin. Anesth.* 90:111221. 10.1016/j.jclinane.2023.111221 37515876

