# Supplementary material for: Association between preoperative sarcopenia and postoperative delirium in older patients undergoing gastrointestinal cancer surgery
Source: Front Aging Neurosci. 2024 Jul 31;16:1416569. doi: 10.3389/fnagi.2024.1416569 (PMC11322050; doi:10.3389/fnagi.2024.1416569)
Supplement: Supplementary file 1 [file Table_1.DOCX]

**Supplement table 1 Collinearity analysis of related variables included in multivariate logistic regression analysis**

| Collinear statistics | | |
| --- | --- | --- |
| Variables | Tolerance | Variance inflation factor |
| Age ≥ 70 years | .819 | 1.221 |
| Preoperative MMSE scores | .942 | 1.062 |
| Serum albumin (g/L) | .794 | 1.259 |
| Risk of malnutrition, n (%) | .960 | 1.041 |
| Intraoperative blood transfusion, n (%) | .929 | 1.076 |
| Sarcopenia, n (%) | .901 | 1.110 |

Abbreviations: MMSE: Mini-Mental State Examination
